# Primary Bone Sclerosing Epithelioid Fibrosarcoma: A Case Report and Literature Review

**DOI:** 10.7759/cureus.95566

**Published:** 2025-10-28

**Authors:** Maimona M Weisy, Omar A Habboub, Aaron Han, Nicandro Figueiredo, Kalpana Golani, Ayoub Nahal

**Affiliations:** 1 Medicine, Istinye University, Istanbul, TUR; 2 Pathology, Loyola University Medical Center, Maywood, USA; 3 Neurological Surgery, King’s College Hospital London, Dubai, ARE; 4 Pathology, King’s College Hospital London, Dubai, ARE; 5 Pathology, National Reference Laboratories, Abu Dhabi, ARE

**Keywords:** bone, case study, muc-4, sclerosing epithelioid fibrosarcoma, spine, tumor

## Abstract

Sclerosing epithelioid fibrosarcoma (SEF) is an extremely rare soft tissue sarcoma. Primary involvement of the bone, particularly the spine, is exceptional, with only isolated cases described. We report a very rare case of primary spinal SEF occurring in a 30-year-old male who presented with chronic back pain. MRI revealed a mass involving the thoracic vertebrae. CT-guided biopsy showed a densely sclerotic stroma containing atypical spindle-to-epithelioid cells arranged in cords and nests. Immunohistochemistry demonstrated strong positivity for mucin-4 (MUC4) and epithelial membrane antigen, with negative staining for S100, desmin, and CK AE1/AE3, confirming the diagnosis of SEF. Surgical excision and spinal fixation were initially planned; however, the patient relocated abroad before surgery and instead received high-dose proton-beam radiotherapy alone. He remains under follow-up in 2024 without surgical intervention. This case emphasizes the significance of including primary bone SEF in the differential diagnosis of thoracic spinal masses, particularly in individuals experiencing chronic back pain. Timely identification and precise diagnosis with specific immunohistochemistry are crucial in guiding an appropriate treatment strategy. Our analysis of 38 cases, including our case, showed a male predominance, spinal involvement, and a high MUC4 positivity rate, reinforcing its role as a key diagnostic marker.

## Introduction

Sclerosing epithelioid fibrosarcoma (SEF) was first described by Dr. Meis-Kindblom as a rare and distinctive deep-seated variant of fibrosarcoma typically occurring in adults [1]. This tumor shares molecular alterations with low-grade fibromyxoid sarcomas (LGFMS), with respective incidences of 0.15 and 0.51 cases per million people per year [2]. SEF is a malignant neoplasm displaying a deceptively bland morphology yet exhibiting high rates of local recurrence and distant metastasis [1,3]. The most common sites are the lower extremities or pelvic girdle [4]. Bone is rarely a primary site for SEF, and spinal origin, as seen in our patient, is even rarer [5]. In this case review, we report a primary thoracic vertebral SEF, its diagnostic challenges, and treatment strategies.

## Case presentation

**Figure 1:**
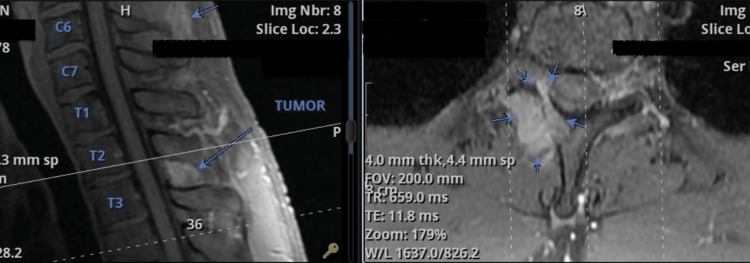
MRI (sagittal T1 with contrast) of the low cervical and thoracic segment of the spine showing an osteolytic lesion with its epicenter in the right lamina and inferior facet of the T2 vertebra. The lesion slightly invaded the right neural foramen, near the exiting right T2 nerve root, posteriorly to the spinous process of the C6 vertebra.

**Figure 2:**
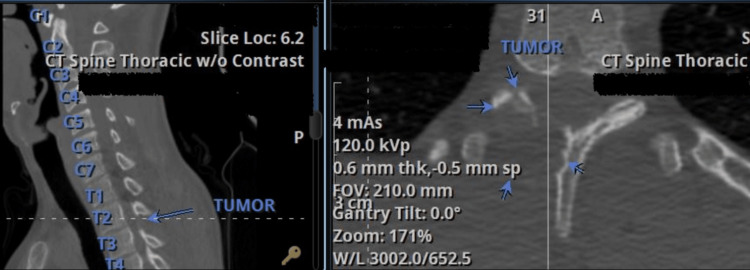
Contrast-enhanced CT showing an osteolytic lesion centered in the right T2 lamina and inferior facet, with slight extension into the right neural foramen near the exiting T2 nerve root.

A 30-year-old Polish national and resident of the United Arab Emirates (UAE) presented with chronic mid-back pain in the cervical and thoracic area. MRI revealed an osteolytic lesion with its epicenter in the right lamina and inferior facet of the T2 vertebra. The tumor had slight invasion into the right neural foramen near the existing right T2 nerve root (Figure 1). MRI also revealed a second oval-shaped enhancing focus adjacent to the main lesion. Although this raised the possibility of a distinct tumor component such as a small hemangioma or vascular malformation, the patient’s age, lesion location, and CT characteristics were felt to be more suggestive of an osteoblastoma. Features on CT, along with age and location, pointed more toward an osteoblastoma (Figure 2). No additional systemic imaging was pursued, as image-guided biopsy was recommended for definitive diagnosis.

**Figure 3:**
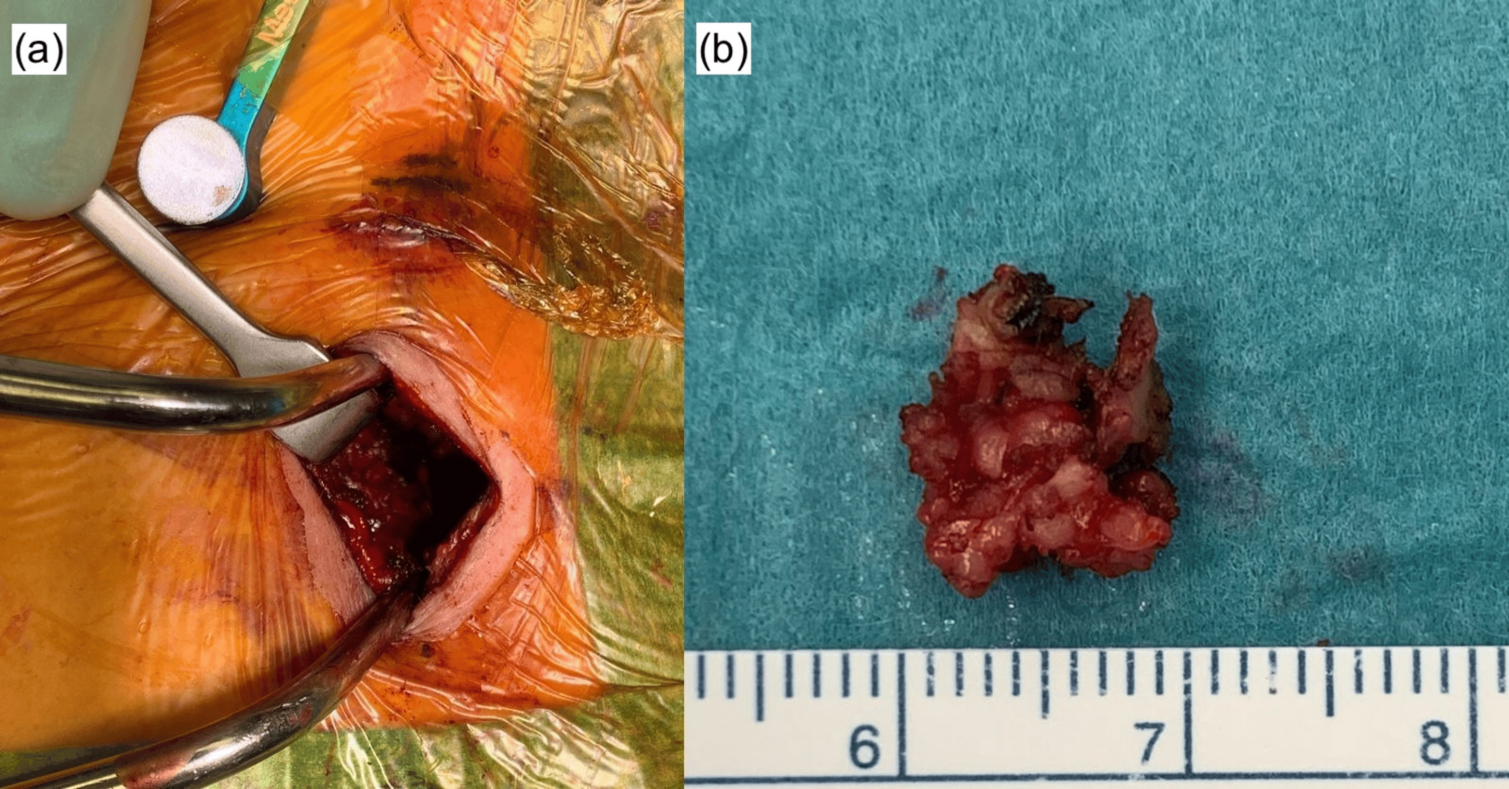
(a) Posterior thoracic T2-3 approach for three-dimensional image-guided biopsy. (b) An irregular fibrous nodule can be seen.

A targeted mini-open spinal biopsy was performed under general anesthesia with intraoperative neuromonitoring to obtain tissue for histological confirmation. Using a posterior right paravertebral T2-3 approach, the neurosurgical team employed intraoperative three-dimensional (3D) imaging (O-arm 2) and neuronavigation (StealthStation S8, Medtronic®, USA) to precisely localize the lesion. The specimen was submitted for frozen section analysis (Figure 3), which revealed only fibrous tissue and was reported as deferred. The postoperative course was uneventful, and the patient was discharged on the first day following surgery.

**Figure 4:**
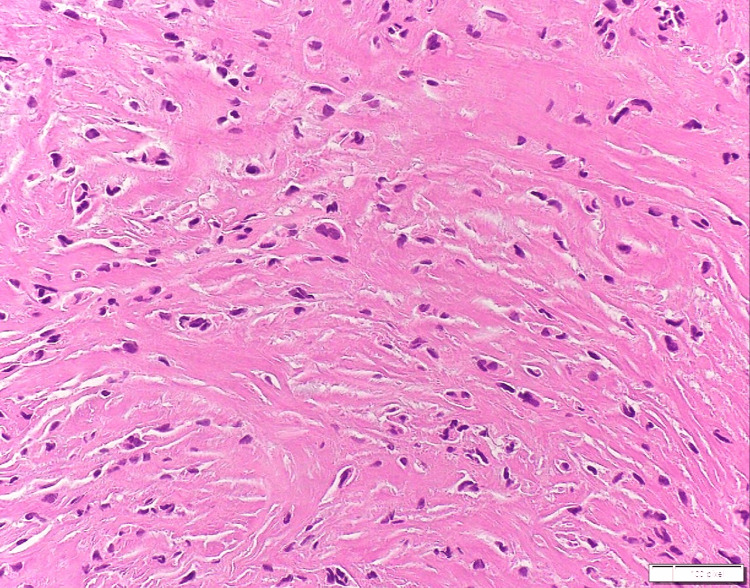
Paucicellular infiltrate of atypical hyperchromatic epithelioid cells embedded in a sclerotic stroma (hematoxylin and eosin stain, 400×).

Permanent histology showed fragments of densely sclerotic fibrous tissue containing atypical spindle and occasional epithelioid cells. These cells displayed irregular, elongated, hyperchromatic nuclei and were arranged haphazardly within a fibrous stroma with focal nuclear crowding. No significant pleomorphism, necrosis, or mitotic activity was identified. The lesion infiltrated adjacent skeletal muscle, and no osteoid or bone matrix was present (Figure 4).

**Figure 5:**
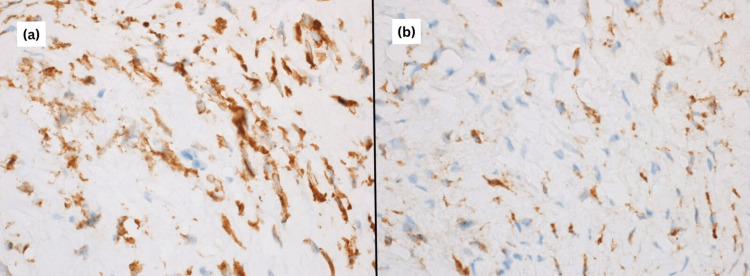
(a, b) Mucin-4 antibody (Cell Marque; clone 8G7) shows strongly positive expression in the cytoplasm of the spindle tumor cells.

Immunohistochemical profiling demonstrated strong cytoplasmic positivity for mucin-4 (MUC4), while staining for CK AE1/AE3, S100, Desmin, SOX10, smooth muscle actin, and β-catenin was negative (Figure 5). Taken together with histomorphology, these findings supported a diagnosis of SEF.

Following biopsy-confirmed SEF, surgery was planned using a posterior cervicothoracic microsurgical approach with decompression (partial laminectomy, facetectomy, and spinous process removal at T2-T3), tumor resection (subtotal or gross total depending on intraoperative findings), excision of a C6 spinal lesion, posterior fixation from T1 to T5 using pedicle screws under neuronavigation, intraoperative 3D imaging, and neuromonitoring. Before the procedure could proceed, however, the patient relocated abroad and instead underwent high-dose proton-beam radiotherapy without surgical intervention, according to the most recent follow-up in 2024.

## Discussion

Sarcomas comprise a diverse group of mesenchymal malignancies characterized by varied clinical presentations and molecular signatures. Fibrosarcoma represents a small fraction of these tumors [6], and SEF is an exceptionally rare variant [2]. Primary osseous involvement, particularly in the spine, is exceedingly uncommon. This case highlights the diagnostic challenges posed by spinal SEF and underscores the importance of recognizing this entity within the spectrum of primary bone tumors [4].

SEF is known to behave more aggressively than its counterpart, LGFMS, which typically carries a lower risk of recurrence and disease-related mortality [2]. Diagnostic recognition of SEF has improved significantly over the past decade with the advent of advanced immunohistochemical techniques, particularly the identification of strong cytoplasmic MUC4 expression, which has emerged as a highly sensitive and specific marker [7].

Molecular testing using reverse transcription polymerase chain reaction and fluorescence in situ hybridization to detect *EWSR1* and *CREB3L1* rearrangements can further support the diagnosis of primary osseous SEF [5,8]. Importantly, however, negative MUC4 staining, or the absence of *EWSR1*/*FUS* gene fusions, does not exclude SEF, as a subset of tumors may instead harbor alternative fusions involving *YAP1* or *KMT2A* [9,10].

**Figure 6:**
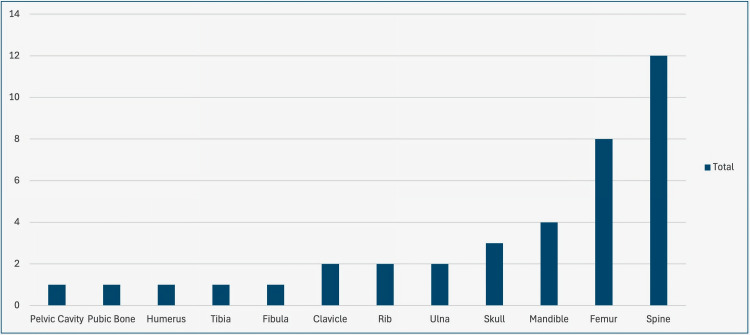
Distribution of cases by anatomic location.

Our literature review identified 38 cases of primary bone SEF to date, including the present one, with a mean age of ~40 years (range = 8-73 years) (Table 1). The appendicular skeleton was the most frequently affected site (14 cases; 38%), followed by the spine (12 cases; 30%), with the remaining cases involving other axial locations such as the trunk, mandible, and skull (Figure 6). Within the 12 spinal cases, the distribution was equal among males and females. Within the male cases, five out of six were in the thoracic vertebrae. Within the female cases, four out of six were found in the lumbar vertebrae. Of the 37 cases tested for MUC4, 34 (92%) were positive, reinforcing its value as a diagnostic marker in both SEF and LGFMS [7].

**Table 1:** Literature review data of 38 cases of primary bone SEF to date. Cases 1-8 [12], case 9 [13], cases 10-18 [11], cases 19-24 [5], cases 25-30 [4], case 31 [14], cases 32-34 [15], 35 [16], case 37 [17], and case 38 [18]. SEF = sclerosing epithelioid fibrosarcoma; MUC4 = mucin-4; EMA = epithelial membrane antigen

Case	Age	Sex	Site of the lesion	Size (cm)	Immunohistochemistry
1	58	F	Femur	5.2	MUC4 (+), SATB2 (–), EMA (–)
2	51	F	Femur	9.6	MUC4(–), SATB2 (–), EMA (–)
3	73	M	Ulna	4	MUC4 (+), SATB2 (–), EMA (–)
4	53	M	Femur	2.8	MUC4 (+), SATB2 (–)
5	25	M	Humerus	11.5	MUC4 (+), SABT (+), EMA (+)
6	66	F	C6	4.2	MUC4 (+), SATB2 (-), EMA (+)
7	31	M	Ulna	3	MUC4 (–), SATB2 (–), EMA(+)
8	43	M	Second rib (R)	8	MUC4 (+), SATB2 (-), EMA (–)
9	64	M	T1-T2	5	MUC4 (+)
10	23	M	Mandible (L)	5.3	MUC4 (+), SATB2 (focal)
11	14	M	Maxilla (L)	5.8	MUC4 (+), SATB2 (focal), Ki67 (20%), EMA (–)
12	36	M	Third rib (L)	7	MUC4 (+), SATB2 (–), Ki67 (10%), EMA (focal)
13	59	F	Tibia	9	MUC4 (+), SATB2 (diffused), Ki67 (10%)
14	71	M	Clavicle	N/A	MUC4 (+), SATB2 (focal)
15	22	F	C6-T1	2.5	MUC4 (+), EMA (–)
16	51	F	L3-S1	2.5	MUC4 (+), SATB2 (–), Ki67 (5-10%), EMA (–)
17	15	F	Femur	7.7	MUC4 (+), SATB2 (Pathcy), Ki67 (5–10%), EMA (focal)
18	60	M	Mandible (L)	N/A	MUC4 (+), SATB2 (–)
19	23	F	Femur	5.7	MUC4 (+), SATB2 (–)
20	8	M	Femur	N/A	MUC4 (+), SATB2 (–)
21	47	M	T vertebrae	N/A	MUC4 (+), SATB2 (–)
22	52	F	Skull	6	MUC4 (+), SATB2 (–)
23	28	M	Mandible	3	MUC4 (+), SATB2 (–)
24	51	F	Femur	5.5	MUC4 (+), SATB2 (–)
25	42	M	C7	7.6	MUC4 (+)
26	29	F	L1	7	MUC4 (+)
27	44	F	L5	3.9	MUC4 (+)
28	50	M	T11	5	MUC4 (+)
29	28	M	T12	4.5	MUC4 (+)
30	41	F	L4	5.5	MUC4 (+)
31	46	M	Pubic bone	N/A	MUC4 (+)
32	26	F	Femur	4.2	MUC4 (+), SATB2 (–), EMA (–), Ki-67 (<3%)
33	16	M	Clavicle	6.5	MUC4 (+), SATB2 (–)
34	47	F	Temporal bone	2.5	MUC4 (+)
35	45	F	Pelvic cavity	13	MUC4 (–), EMA (+)
36	30	M	T2-T3	N/A	EMA (+), MUC4 (+)
37	52	M	Mandible	1.9	MUC4 (+)
38	19	M	Fibula	3	N/A

Negative SATB2 staining with MUC4 positivity has also been shown to be a valuable marker in the diagnosis of SEF as opposed to osteosarcoma [5,11,12]. In our review of the 24 cases that tested for both, 16 cases stained both positive for MUC4 and negative for SATB2.

## Conclusions

We report a rare case of SEF arising in a thoracic paravertebral location, an uncommon osseous manifestation of this already rare sarcoma. Diagnostic precision for SEF has markedly improved in recent years, with MUC4 now recognized as a highly sensitive and specific immunohistochemical marker. In addition, detection of gene fusions involving *EWSR1* and *CREB3L1* further supports molecular confirmation of primary bone SEF. Given its subtle morphology and overlapping features with other spindle cell neoplasms, accurate classification of primary bone SEF relies on both immunohistochemistry and molecular testing. This entity should be included in the differential diagnosis of paravertebral lesions, particularly in patients presenting with persistent mid-back pain in the cervical or thoracic spine.
